# Evaluating prescribing competencies covered in a Canadian-accredited undergraduate pharmacy program in Qatar: a curriculum mapping process

**DOI:** 10.1186/s12909-020-02109-1

**Published:** 2020-08-06

**Authors:** Oraib Abdallah, Rwedah Anwar Ageeb, Wishah Hamza Imam Elkhalifa, Monica Zolezzi, Alla El-Awaisi, Mohammad Issam Diab, Ahmed Awaisu

**Affiliations:** 1 Mental Health Services, Hamad Medical Corporation, PO Box 3050, Doha, Qatar; 2 College of Pharmacy, QU Health, Qatar University, PO Box 2713, Doha, Qatar; 3 Pharmacy Department, Hamad General Hospital, Hamad Medical Corporation, PO Box 3050, Doha, Qatar

**Keywords:** Competencies, Curriculum mapping, Non-medical prescribing, Pharmacists prescribing, Prescribing

## Abstract

**Background:**

The objective of this study was to evaluate the existing Bachelor of Science in Pharmacy [BSc (Pharm)] curriculum at Qatar University College of Pharmacy (QU CPH), for addressing international prescribing competencies.

**Methods:**

The Australian National Prescribing Service (NPS MedicineWise) *Competencies Required to Prescribe Medicines* framework (the Prescribing Competencies Framework) was used in the BSc (Pharm) curriculum mapping process. The NPS MedicineWise Prescribing Competencies Framework outlines seven competency areas that are essential for pharmacist prescribing. The first mapping activity assessed the learning outcomes (LOs) of 62 courses within the BSc (Pharm) curriculum for covering and addressing the NPS MedicineWise competencies. The second mapping activity involved matching the LOs identified to address the NPS MedicineWise prescribing competencies, to the 2017 Association of Faculties of Pharmacy of Canada (AFPC) educational outcomes, on which the QU CPH BSc (Pharm) program is based. The AFPC educational outcomes address seven key program-level learning outcomes.

**Results:**

The QU CPH BSc (Pharm) curriculum addresses most of the prescribing competencies listed in the NPS MedicineWise Prescribing Competencies Framework. However, gaps were identified in the curricular content and in the LOs that were related, but not restricted, to the following: electronic prescribing, physical examinations/preparing patients for investigations, and policies/procedures and quality assurace related to prescribing. Other gaps identified include legislative and workplace requirements for obtaining consent to access confidential patient's health information.

**Conclusion:**

The curriculum mapping exercise provided evidence that, for the most part, the existing BSc (Pharm) curriculum at QU CPH prepares pharmacy graduates for prescribing. However, there are areas that need better alignment between the taught curriculum and training on prescribing in practice. The results of this study are important to consider if pharmacist prescribing is to be implemented in Qatar.

## Background

Significant advances in the healthcare system have been achieved over the past few decades. For these advances to take place, expanding the scope of practice and the prescribing role of healthcare practitioners has become necessary to ensure efficient healthcare delivery. In this regard, several countries have changed their legislation to introduce non-medical prescribing (NMP) [1]. NMP authorizes non-medical healthcare professionals, such as pharmacists and nurses, to prescribe pharmacological agents that are legislated as “prescription medicines” under legal certification. Nursing community-based prescribing was the first form of NMP implemented in the United Kingdom (UK) in 1992, which paved the way for prescribing by other non-medical practitioners [1]. In 2015, it was estimated that there were 53,572 nurses, 3845 pharmacists, and 689 allied healthcare providers, registered as either supplementary or independent prescribers in the UK [2]. Similarly, several countries including the United States (US), Australia, New Zealand, and Canada have endorsed NMP with varying degrees and levels of prescribing authorities [3, 4]. Currently, there are several models of NMP, including: independent, collaborative, and dependent prescribing models; the latter includes supplementary prescribing, prescribing by protocols, patients group direction, prescribing by formulary, prescribing by patient referral, and repeat prescribing [4].

The pharmacist’s scope of practice has evolved beyond the traditional dispensing of medications. Pharmacists are taking on expanded roles and are increasingly being recognized as the medication management experts of the health care team, including various levels of prescribing [4]. Studies evaluating pharmacist prescribing practice have shown promising results in improving healthcare outcomes [5–9]. A randomized controlled trial which assessed pharmacist-directed dyslipidemia care including prescribing of medications showed three times more patients achieving target lipid levels at six months compared to usual care [5]. A systematic review identified that when pharmacists were provided prescribing authority for managing patients with diabetes, patients achieved a greater reduction in their glycated hemoglobin (HbA_1C_) levels compared to patients who received usual care [6]. Similar studies have also shown a significant impact of pharmacist prescribing in patients with hypertension, chemotherapy adverse event management, and in patients receiving anticoagulation therapy [7–9].

Several prescribing competency frameworks have been described by health-related institutions such as the World Health Organization (WHO), the UK’s Royal Pharmaceutical Society, the Australia’s National Prescribing Service (NPS MedicineWise), and others. The WHO framework consists of a six-step prescribing model, which includes defining the patient’s problem, specifying the therapeutic objectives, verifying the suitability of the prescribing drug (drug choice for a specific indication), writing a prescription, giving information, instructions and warnings to the patients, and monitoring the treatment [10]. The UK’s Royal Pharmaceutical Society Competency Framework for all Prescribers has set 10 competencies describing activities and outcomes that should be demonstrated by a prescriber, such as assessing the patient, considering alternatives, and reaching a shared decision [11]. The Australia’s NPS MedicineWise Competencies Required to Prescribe Medicines (the Prescribing Competencies Framework), published in 2012, is a comprehensive framework outlining seven competency areas (CAs) required for appropriate and safe prescribing practice, of which five are directly related to the prescribing conduct: CA1(Assessment): understands the person and their clinical needs; CA2 (Treatment Options): understands the treatment options and how they support the person’s clinical needs; CA3 (Shared Decision Making): works in partnership with the person to develop and implement a treatment plan; CA4 (Coordination): communicates the treatment plan clearly to other health professionals and; CA5 (Monitors and Reviews): monitors and reviews the person’s response to treatment. Additionally, there are two CAs related to professional behaviors, referred to as “horizontal competency areas” (CAH), which are considered crucial to guide prescribing: CAH1 (Professional): Practices professionally and; CAH2 (Communicates): Communicates and collaborates effectively with the person and other health professionals [12]. Table 1 summarizes these competencies.

**Table 1:** The seven competency areas listed in the Australian National Prescribing Serices (NPS MedicineWise) Competencies Framework

1. **Assessment:** Understands the person and their clinical needs	
2. **Treatment Options:** Understands the treatment options and how they support the person’s clinical needs	
3. **Shared Decision Making:** Works in partnership with the person to develop and implement a treatment plan	
4. **Coordination:** Communicates the treatment plan clearly to other health professionals	
5. **Monitors and Reviews:** Monitors and reviews the person’s response to treatment	
6. **Professional:** Practices professionally^a^	
7. **Communicates:** Communicates and collaborates effectively with the person and other health professionals^a^	

^a^These are two horizontal competency areas that integrate with the other five main competencies

The Table is adapted and summarized from the Australian National Prescribing Services (NPS MedicineWise) Competencies Framework^12^

The establishment of a Bachelor of Science in Pharmacy [BSc (Pharm)] program at Qatar University College of Pharmacy (QU CPH) in 2007 and a post-baccalaureate Doctor of Pharmacy (PharmD) program in 2011, both accredited by the Canadian Council for Accreditation of Pharmacy Programs (CCAP), has facilitated expanded roles for pharmacists in Qatar over the past decade. Being CCAP-accredited, the BSc (Pharm) program at QU CPH was designed to meet the educational outcomes identified by the Association of Faculties of Pharmacy of Canada (AFPC) [13]. These AFPC educational outcomes were developed for all entry-to-practice pharmacy programs in Canada, and include seven major role statements (program learning outcomes) addressing several key competencies that pharmacy graduates should attain by the end of their degree program, including: Care Provider, Communicator, Collaborator, Leader-Manager, Health Advocate, Scholar, and Professional [13].

Despite these advancements in pharmacy education, pharmacist prescribing in Qatar remains limited, mostly as part of collaborative practice agreements in some multidisciplinary care settings, such as anticoagulation clinics [9]. The future implementation of pharmacist prescribing in Qatar should rely on a solid foundation and a high level of competence of pharmacy graduates that qualify them to assume prescribing roles. Accordingly, the aim of this study was to evaluate, through a curriculum mapping process, the adequacy of the BSc (Pharm) program at QU CPH in addressing prescribing competencies. The results will identify gaps related to international prescribing competencies in the curriculum and shall provide an opportunity for reflection on the future implementation of pharmacist prescribing in Qatar.

## Methods

### Selection of prescribing competency framework

After an extensive review of the literature, the Australian NPS MedicineWise Prescribing Competencies Framework [12] was chosen for the mapping process as it provides an extensive description of the prescribing competency standards with evidence examples that facilitated mapping the learning objectives (LOs) of all courses covered in the BSc (Pharm) curriculum at QU CPH. Essentially, the framework details the practice expectations for prescribers, including the knowledge, skills and attitudes required to safely and effectively prescribe medicines. It plays an important role in informing the prescribing practice expectations of eligible registered health professionals and the prescribing curriculum [12]. More details about the framework were provided in the Background section and Table 1.

### Mapping procedure

Copies of the syllabi for all the 2017–18 academic year BSc (Pharm) courses were obtained from the QU CPH electronic course management system (Blackboard^®^). Only courses related to the four-year BSc (Pharm) professional degree program were included in the mapping process, consisting of 62 courses (120 credit hours). General pharmacy and foundation-year courses were excluded from this curriculum mapping. The curriculum mapping process involved two major mapping activities. The first, assessed the LOs outlined within the courses syllabi for covering and addressing the NPS MedicineWise prescribing CAs [12]. The second, matched the relevant LOs identified in the first step to the 2017 AFPC educational outcomes. This is because, the BSc (Pharm) program is accredited by CCAP (Canada) and the curriculum is designed to meet the educational outcomes outlined by the AFPC for all entry-to-practice pharmacy programs in Canada.

A data collection form, composed of a matrix of the prescribing competencies versus the LOs of all courses, was used to facilitate documentation of the mapping process. Appendix in Table 3 illustrates a sample portion of the data collection form, with an example of how the data were extracted in the mapping process. A triangulation process ensured all competencies and LOs were comprehensively outlined. The final mapping process was conducted by a panel of experts comprising three pharmacy professors who were course coordinators, two final year BSc (Pharm) students who were part of the research team, and a research assistant who graduated from the same program, using a modified Delphi technique, consisting of five rounds of review in which the panel assessed the appropriateness of matching the LOs to the CAs. Results were analyzed as a narrative description of the prescribing-related CAs and the identified gaps in the curriculum.

## Results

The majority of the CAs addressed in the Australian NPS MedicineWise prescribing framework were addressed in the QU CPH BSc (Pharm) curriculum [12]. Table 2 illustrates the gaps identified during the mapping process.

**Table 2:**
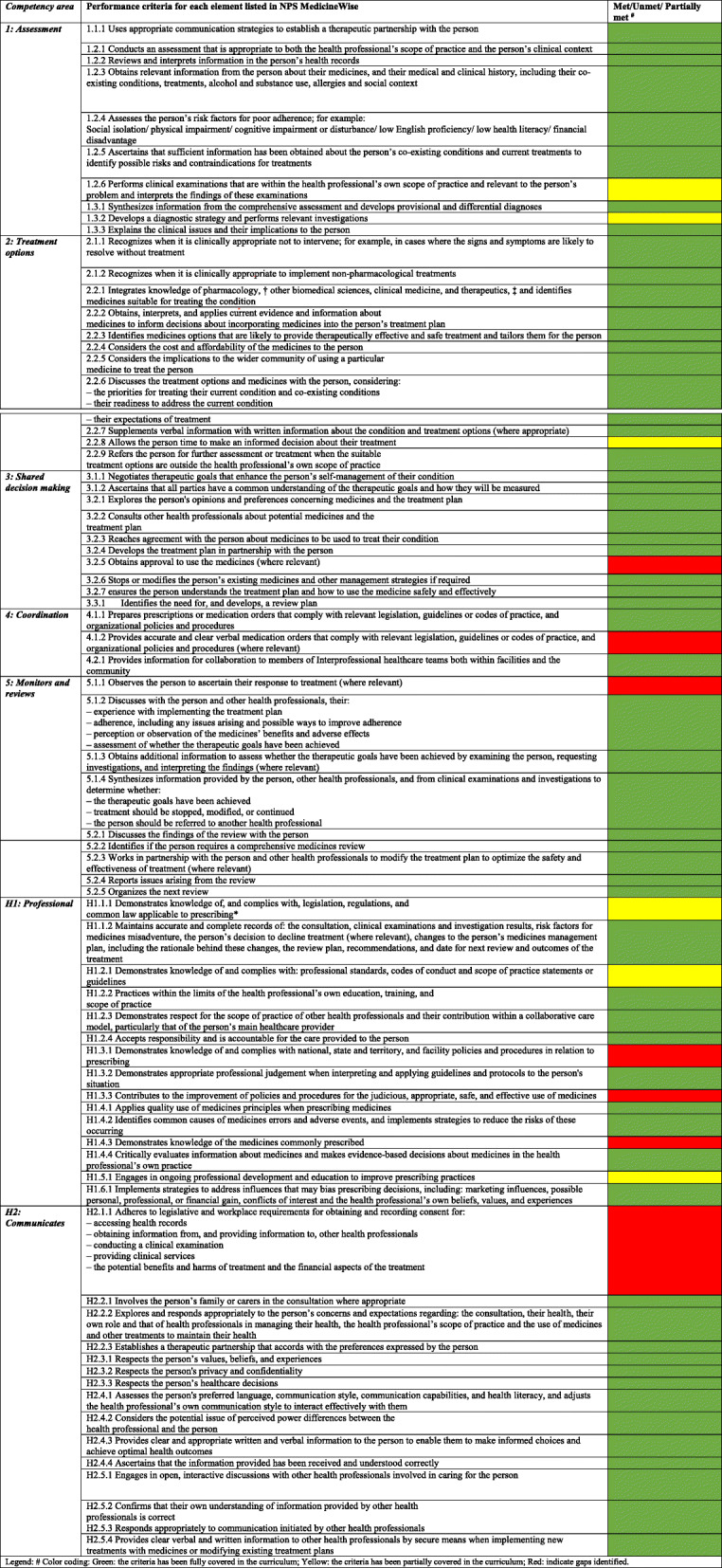
Competencies addressed from the NPS MedicineWise Prescribing Competencies Framework and their coverage in the CPH BSc (Pharm) curriculum

### Competency area 1: Assessment

Most of the performance criteria under this competency were covered in the content of courses in the curriculum. However, two criteria were only partially met, specifically #1.2.6 and #1.3.2. Gaps were identified in addressing the intended LOs for assigning priority to patient examinations, preparing patients for examinations and investigations, and in assessing the clinical relevance during examinations.

### Competency area 2: Treatment options

The Delphi process identified that the LOs related to criteria # 2.2.8 8 that pertains to “allows the person time to make an informed decision about their treatment” were too broad. With this exception, almost all the criteria listed under this CA were adequately covered in the curriculum.

### Competency area 3: Shared decision making

In this competency area, gaps were primarily identified in the content related to criteria # 3.2.5 that pertains to obtaining approval from people to the use of medicines. This particular point was not matched with any of the LOs in the curriculum.

### Competency area 4: Coordination

This competency was mostly covered in the content of courses in the pharmacy curriculum. However, the performance criteria # 4.1.2 related to providing accurate and clear verbal medication orders when prescribing was not matched with any of the LOs. Although this is a basic element that is expected to be covered in any school of pharmacy, the team recognized that it might be delivered but not listed as part of the LOs in the intended curricula.

### Competency area 5: Monitors and reviews

In this particular competency that concerns reviewing and monitoring the patient response to treatment, a gap was identified in criteria #5.1.1 related to observing the person to ascertain their response to treatment.

### Horizontal competency area H1: Professional

Several gaps were identified in the LOs addressing professional practice. Knowledge of legislation, regulations, common law applicable to prescribing, professional standards, codes of conduct and scope of practice statements or guidelines related to prescribing in addition to engaging the students in ongoing professional development and opportunities to improve prescribing practices were partially met in some courses, but were not mainly related to prescribing. Additionally, no course covered the content related to policies and procedures and quality assurance in relation to prescribing.

### Horizontal competency area H2: Communicates

Most of the competency criteria related to effective communication and collaboration with patients and other healthcare providers were covered in the LOs of courses in the curriculum with the exception of criteria #H2.1.1, which was related to legislative and workplace requirements for obtaining and recording consent for accessing and divulging patient’s health information, conducting clinical examination, providing clinical services, the potential benefits and harms of treatment and the financial aspects of the treatment.

## Discussion

The curriculum mapping process undertaken revealed that for the most part, the LOs of the courses in the QU CPH BSc (Pharm) program adequately addressed competencies that are required for a rational, safe and effective prescribing conduct. It further identified gaps in the curriculum where some competencies are not covered or are only partially addressed. Although the curriculum addresses most of the prescribing competencies outlined in the international prescribing frameworks, there are areas in need of improvement, further development, and in-depth coverage. Prescribing competencies are needed to ensure consistent and conventional prescribing process and should be an integral part of curricula that qualify healthcare professional students for undertaking prescribing roles.

Prescribing in undergraduate education has been evaluated in studies conducted primarily at medical schools. For instance, an international multicenter cross-sectional study that aimed to evaluate final-year medical students across Europe for acquiring adequate prescribing competencies before graduation, demonstrated an overall lack of fundamental prescribing competencies among medical students in 15 European countries [14]. Keijsers and colleagues aimed to explore and compare the knowledge and skills of undergraduate medical and pharmacy students in pharmacology and pharmacotherapy to serve as a starting point for constructing multi-disciplinary programs for better prescribing [15]. The study found that the pharmacy students had better knowledge in pharmacology than the medical students, whereas the medical students demonstrated better skills in writing prescriptions, while both groups had similar knowledge in pharmacotherapy [15]. In Warholak and colleagues study, pharmacy students demonstrated better skills than medical students in identifying errors in prescriptions, which is crucial for ensuring safety when prescribing [16]. Ross and colleagues developed comprehensive LOs for teaching prescribing skills in medical schools, and emphasized on the need for reducing medication errors and ensuring efficient communication in order to improve prescribing competencies [17].

This study presented a thorough mapping of QU CPH BSc (Pharm) curriculum. It deconstructed the intended curriculum relative to the Australian NPS MedicineWise Prescribing Competencies Framework [12]. The mapping process demonstrated the high capacity of the intended curriculum to addressing international competencies required for rational, safe and effective prescribing practice. However, some important gaps in educational LOs were identified, particularly those addressing content related to performing clinical or physical examinations. Although various aspects related to patients’ clinical assessment were covered within some courses in the QU CPH BSc (Pharm) curriculum, such as *Patient Assessment, Structured Practical Experiences in Pharmacy (SPEP), Interpretation of Laboratory Data, and Professional Skills*, assigning priority to examinations, preparing patients for examinations and investigations, and assessing the clinical relevance during examinations, were prescribing skills only partially addressed or lacking in the curriculum.

Several studies in relation to clinical examinations in the undergraduate pharmacy curriculum have reported similar findings. A study across schools and colleges of pharmacy in the US reported a significant variability and depth in the content and extent of patient assessment skills within the pharmacy curricula [18]. As pharmacy services are focusing on direct patient outcomes, Bolesta and colleagues highlighted that conducting patient assessments are essential in the pharmacy curricula to address the evolving role of pharmacists [19]. In the study by Chua and colleagues, a group of pharmacists described their experience integrating patient assessment skills within their practice as most valuable in the absence of physicians, when being the last point of contact prior to discharge, and when delegated prescribing authority [20]. The findings of these studies may reflect a lack of responsiveness in the undergraduate pharmacy curriculum to the evolving role of pharmacists worldwide.

Gaps were also identified in the performance criteria related to the use of electronic systems for prescribing purposes. Exposing students to electronic health records and other similar electronic systems through an educational institution is challenging, and requires technological resources to facilitate and support the delivery [21]. Experiential training in different healthcare settings allows for introducing students to electronic systems and patient records. The depth in which electronic systems is covered in the curriculum is heavily influenced by the extent to which it is covered during the students’ experiential education. Because electronic systems vary among the different healthcare settings and are not inter-connected, most learning relies on site preceptors’ experience and the enabling active instructional capacity of the site [21]. The UK National Working Group performed a study that aimed at incorporating electronic patient records into the curriculum of pharmacy, medicine, nursing and midwifery. They identified a group of competencies and LOs that should be delivered to students in the context of electronic patient records, such as the use of electronic systems for referring to patient records for planning and reviewing clinical care and decision making, for communicating effectively with other healthcare providers, and for reviewing and documenting treatment plans. Incorporating these LOs within the QU CPH BSc (Pharm) curriculum may help address these identified gaps in prescribing competencies.

Another gap identified was lack of content related to policies and procedures, and the improvement of these policies and procedures in relation to prescribing. During experiential training, some students may be introduced to prescribing protocols followed by the hosting experiential setting. Because pharmacist prescribing in Qatar is still very limited, policies relating to prescribing may still be under development and many students may not have access to them or exposure to prescribing-related policies during their experiential education. Although there are some collaborative prescribing agreements that allow pharmacists to have a degree of prescriptive authority in some hospitals (for example, in anticoagulation and heart failure clinics), there is no nationwide legal authority for pharmacists to prescribe medicines in Qatar. In addition, pharmacists in community pharmacies are only allowed to recommend over-the-counter medications, which limits the access to important primary prevention medications and limit the accessibility of the public to important health assessment and screening services that could be offered by pharmacists [22]. Experiences in several countries suggest that curricular changes must be accompanied with changes in nationwide policies in order to move the agenda of pharmacist prescribing forward.

Through a curriculum mapping process, this study has provided an opportunity to highlight many action items that should be sent to the QU CPH Curriculum Committee reflecting on the findings and recommending initiatives for improving the undergraduate curriculum in areas related to prescribing competencies. A summary of the recommendations for improving the curriculum based on the gaps identified include the incorporation of content in the curriculum related clinical examination, electronic records, and policies and procedures. Appropriate contents that are constructively aligned with the courses’ LOs should be developed and introduced into the syllabi. Accordingly, valid and objective learning assessment strategies to assess students’ progress toward the achievement of the intended LOs for the prescribing competencies should also be developed.

A limitation in the curriculum mapping process is that we only assessed the LOs of the courses syllabi for addressing prescribing competencies. Furthermore, the LOs listed under individual lectures within each course were not evaluated to assess if they were similar and consistent with those of the intended curriculum. Perhaps the fact that four of the panel members were faculty members who were involved in teaching several of the courses has mitigated these limitations. For this reason, there were other research activities that were performed to assess the prescribing competencies in the curriculum by the same research team members (e.g. we conducted surveys among students, faculty members, and recent graduates to assess their perspective regarding prescribing competencies and pharmacist prescribing in general), which may add strength to the current findings.

## Conclusion

The curriculum mapping provided evidence that the BSc (Pharm) curriculum at QU CPH for the most part adequately prepares pharmacy students to achieve prescribing competencies. However, improvement in some components will be needed in order to qualify pharmacy students for undertaking prescribing roles, particularly in relation to patient assessment competencies as these skills are considered essential for diagnosis, prescribing, and monitoring the efficacy and safety of medications prescribed. Therefore, the findings of this study along with the other related studies could help in the foundational basis of future implementation of pharmacist prescribing in the country. The curriculum mapping is vital in informing the prescribing practice expectations of pharmacy graduates and future prescribing curriculum.

## Data Availability

Not applicable.

## Appendix

**Table 3:** Sample portion of a detailed report for the curriculum mapping based on NPS MedicineWise for competency area number 1

*Competency area 1: Assessment*			
	Learning Objectives of CPH courses that match NPS MedicineWise competencies	CPH courses	Gaps /notes
Element 1.1 **Establishes a therapeutic partnership with the person and a collaborative** **relationship with other health professionals**	**Performance criteria 1.1.1 Uses appropriate communication strategies to establish a therapeutic partnership with the person**		
	• Develop and maintain professional, collaborative relationships required for patient care (*linked to AFPC KC 2017: CP 1.2, CL1.1)*	Pharmacy and health care (231), Integrated case based learning (391, 490), Drugs in sport (444) Rx elective (545), Structured Practical experiences in pharmacy - SPEP courses (330, 430,530,531, 532,533), Professional skills (340,341,440,441)	“Drug in sports – PHAR 444” is an elective course, not delivered to all students.
	• Work collaboratively with the patient and his/her health care professionals to provide care and services that facilitate management of the patient’s health needs *(linked to AFPC KC: CL1)*	Pharmacy and health care (231), Drugs in sport (444), prof. Skills series courses (241, 340,341,440,441), Structured Practical experiences in pharmacy - SPEP courses (330, 430,530,531, 532,533	
	• Recognize and begin to demonstrate the fundamental communication skills required of a pharmacist. *(linked to AFPC KC: CM1 and CM2)*	Professional skills course (241)	
	• Communicate verbally and non-verbally with others *(linked to AFPC KC: CM1.1 and CM 1.7 and CM2)*	Professional skills courses (240, 241,340, 341, 440, 441) Pharmacy and health care (231) Pharmacy research, evaluation and presentation skills (305, 306,406, 505,506) Rx elective (446), Structured Practical experiences in pharmacy - SPEP courses (330, 430,530,531, 532,533), Patient assessment Laboratory (361,362)	
	• Actively make their expertise available to others and willingly agree to share relevant information, using language that can be understood by all *(linked to AFPC KC: CL2)*	Professional skills courses (240, 241,340, 341, 440, 441), Pharmacy research, evaluation and presentation skills (406), Rx elective (446,545), Drugs in Sport (444), Structured Practical experiences in pharmacy - SPEP courses (330, 430,530,531, 532,533)	
	• Understand the importance of establishing a therapeutic alliance with patients*. (linked to AFPC KC: CL1.1)*	Integrated case-based learning (390)	

Legend: CPH: College of Pharmacy, NPS: National Prescribing Service, AFPC: 2017 Association of Faculties of Pharmacy of Canada (AFPC) educational outcomes, KC: Key competencies, CP: care provider CL: collaborator, CM: communicator

## Abbreviations

AFPC: Association of Faculties of Pharmacy of Canada
BSc (Pharm): Bachelor of Science in Pharmacy
CAH: Horizontal competency areas
CAs: Competency areas
CCAP: Canadian Council for Accreditation of Pharmacy Programs
LOs: Learning outcomes
MSC: Medical Schools’ Council
NPS: National Prescribing Services
QU CPH: Qatar University College of Pharmacy
SPEP: Structured Practical Experiences in Pharmacy
UK: United Kingdom
US: United States
WHO: World Health Organization
